# Evaluation of Flow Cytometry for Cell Count and Detection of Bacteria in Biological Fluids

**DOI:** 10.1128/spectrum.01830-21

**Published:** 2022-02-23

**Authors:** N. Dossou, I. Gaubert, C. Moriceau, E. Cornet, S. le Hello, D. Malandain

**Affiliations:** a CHU de Caen Normandie, Department of Microbiology, Caen, France; b CHU de Caen Normandie, Department of Haematology, Caen, France; Houston Methodist Hospital

**Keywords:** biological fluids, cell count, detection of bacteria, flow cytometry

## Abstract

The analysis of biological fluids is crucial for the diagnosis and monitoring of diseases causing effusions and helps in the diagnosis of infectious diseases. The gold standard method for cell count in biological fluids is the manual method using counting chambers. The microbiological routine procedures consist of Direct Gram staining and culture on solid or liquid media. We evaluate the analytical performance of SYSMEX UF4000 (Sysmex, Kobe, Japan) and Sysmex XN10 (Sysmex, Kobe, Japan) in comparison with cytological and microbiological routine procedures. A total of 526 biological fluid samples were included in this study (42 ascitic, 31 pleural, 31 peritoneal, 125 cerebrospinal, 281 synovial, and 16 peritoneal dialysis fluids). All samples were analyzed by flow cytometry and subsequently processed following cytological and/or microbiological routine procedures. With regard to cell counts, UF4000 (Sysmex, Kobe, Japan) showed a performance that was at least equivalent to those of the reference methods and superior to those of XN10 (Sysmex, Kobe, Japan). Moreover, the bacterial count obtained with UF4000 (Sysmex, Kobe, Japan) was significantly higher among culture or Direct Gram stain positive samples. We established three optimal cutoff points to predict Direct Gram stain positive samples for peritoneal (465.0 bacteria/μL), synovial (1200.0 bacteria/μL), and cerebrospinal fluids (17.2 bacteria/μL) with maximum sensitivity and negative predictive values. Cell count and detection of bacteria by flow cytometry could be used upstream cytological and microbiological routine procedures to improve and accelerate the diagnosis of infection of biological fluid samples.

**IMPORTANCE** The analysis of biological fluids is crucial for the diagnosis and monitoring of diseases causing effusions and helps in the diagnosis of infectious diseases. The possibility of carrying out cytological and microbiological analyses of biological fluid samples on the same automated machine would simplify the sample circuit (addressing the sample in a single laboratory, 24/7). It would also minimize the quantity of sample required. The performance of cytological and microbiological analysis by the flow cytometer UF 4000 (Sysmex, Kobe, Japan) has never been evaluated yet. This study has shown that bacterial count by flow cytometry using UF4000 (Sysmex, Kobe, Japan) could be used upstream of microbiological routine procedures to improve and to accelerate the diagnosis of infection of biological fluid samples.

## INTRODUCTION

The analysis of biological fluids is crucial for the diagnosis and monitoring of diseases causing effusions and helps in the diagnosis of infectious diseases. The gold standard method for cell count in biological fluids is the manual method using counting chambers (1). The current REMIC recommendations for microbiological procedures consist of Direct Gram staining (DGS), culture on solid media, liquid media, and/or incubation in blood culture bottles. These manual methods require significant expertise and technical time.

Cytological and microbiological analysis of effusion fluids, cerebrospinal fluids, and synovial fluids should be performed promptly because of the rapid degradation of cells, especially neutrophils. In addition, the results of these analyses are important tools for clinical decision-making, particularly in the case of infectious diseases, where the early initiation of appropriate anti-infectious treatment results in a better prognosis (2–4).

The automation of these analyses has several advantages over manual methods, due to its speed and the lack of a need for sample pretreatment. Being operable 24/7 and offering reliable analysis, the SYSMEX XN10 (Sysmex, Kobe, Japan) and the SYSMEX UF4000 (Sysmex, Kobe, Japan) machines could be an interesting alternative to manual methods.

The aim of this study was to evaluate the analytical performance of the SYSMEX UF4000 (Sysmex, Kobe, Japan) and SYSMEX XN10 (Sysmex, Kobe, Japan) as methods for the cytological analysis of different body fluids in comparison to manual methods: cell count in KOVA counting chambers and differential leukocyte count after staining with May Grunwald Giemsa (MGG).

The secondary aim of this study was to evaluate the analytical performance of the SYSMEX UF4000 (Sysmex, Kobe, Japan) as a method for the microbiological analysis in comparison with DGS and/or the conventional culture on solid media, liquid media and enrichment broth.

## RESULTS

### Comparison of the performance of cytological analysis methods.

A total of 189 biological fluids samples were analyzed by flow cytometry (FCM) on the SYSMEX UF4000 (Sysmex, Kobe, Japan) and the SYSMEX XN (Sysmex, Kobe, Japan). They were subsequently processed by the routine cytological procedures (white blood cell and red blood cell count in KOVA counting chambers, manual differential leukocyte count after staining with MGG). Seven samples were excluded due to a lack of clinical or biological information. Finally, 182 samples originating from 151 patients were included in this part of the study: 42 ascitic fluids (AF), 31 pleural fluids (PF), 31 peritoneal fluids (PRF), 16 peritoneal dialysis fluids (PDF), 30 synovial fluids (SF), and 32 cerebrospinal fluid shunts (CSF). The mean age was 60 years (interquartile range [IQR]: 22-66) and the sex ratio was 1.43. The characteristics of the included patients are shown in Table 1.

**TABLE 1:** Characteristics of included patients for comparison of the performance of cytological analysis methods

Variable	All fluids	AF^*a*^	PF^*b*^	PRF^*c*^	PDF^*d*^	SF^*e*^	CSF^*f*^
Total	182	42	31	31	16	30	32
Age (mean ± SD)	60.47 ± 19.91	64.76 ± 15.49	63.84 ± 17.34	49.10 ± 24.78	51.56 ± 29.23	69.17 ± 12.80	58.91 ± 16.18
Sex ratio	1.43	2.23	1.07	1.38	1.29	2.33	0.78
Hematologic malignancy, *n* (%)	13 (7.14%)	7 (16.67%)	5 (16.13%)	1 (3.23%)	0 (0.00%)	0 (0.00%)	0 (0.00%)
Antimicrobial treatment, *n* (%)	59 (32.42%)	11 (26.19%)	6 (19.35%)	14 (45.16%)	8 (50.00%)	5 (16.67%)	15 (45.88%)

a AF: ascitic fluid.

b PF: pleural fluid.

c PRF: peritoneal fluid.

d PDF: peritoneal dialysis fluid.

e SF: synovial fluid.

f CSF: cerebrospinal fluid shunt.

**(i) Cell count.** The three methods compared in this study have different limits of quantification for red blood cell (RBC) and white blood cell (WBC) counts; they are shown in Table 2. Red blood cell counts obtained by FCM using the SYSMEX XN (Sysmex, Kobe, Japan) were rounded to 1,000/μL.

**TABLE 2:** Limits of quantification for red blood cell and white blood cell counts obtained by automated methods UF and XN, and by standard procedure

Blood cell	Sysmex XN10	Sysmex UF4000	KOVA
Red blood cell	0–10×10^6^/μL	15–100,000/μL	0–1,000/μL
White blood cell	0–5,000 ×10^3^/μL	2–10,000/μL	0–1,000/μL

**FIG 1:**
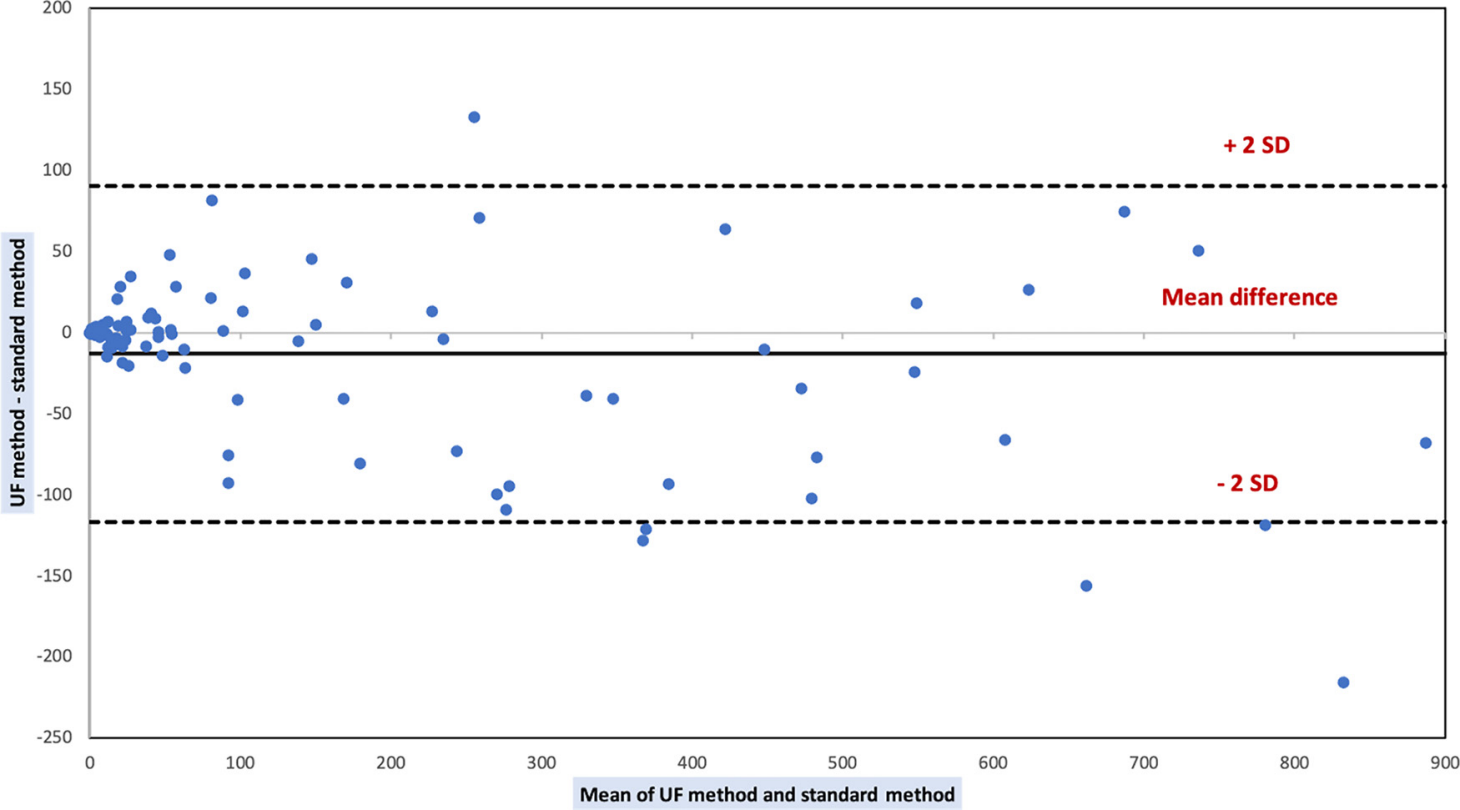
Bland Altman plot of differences between red blood cell counts by UF and standard method. *SD = standard deviation.

Comparison results of the automated methods to the KOVA standard method for RBC counts are shown in Table 3 and in Fig. 1. The comparison was performed on 96 samples. Eighty-six samples were excluded from the comparative analysis because standard method results were outside the limits of quantification (>1,000 red blood cells/μL). They also had results greater than >1,000 RBC/μL with the XN method, but 3 had results <1,000 RBC/μL with the UF method: 332/μL, 807.9/μL and 929.7/μL, respectively. The first two were pleural fluids taken from patients with pleural effusions, with no reported history of hematological malignancy or antibiotic therapy. The last was a peritoneal fluid sample taken from a patient with peritonitis symptoms and who was on broad-spectrum antibiotherapy.

**TABLE 3:** Comparison of red blood cell counts obtained by XN and UF methods to the standard method^*a*^

Variable	Sysmex XN	Sysmex UF
Mean difference	46	−15.16
Lower limit of agreement	−926	−154.14
Upper limit of agreement	1017	123.82
Spearman’s correlation coefficient	0.5426 (*P* < 0.00001*)	0.9616 (*P* < 0.00001*)
Regression equation	Y = 0.218x + 125.32	Y = 1.0341x + 9.7765
Paired Wilcoxon test (bilateral)	*P* = 0.00089*	*P* = 0.11088

a All statistical tests were carried out in comparison with standard method (Red blood cell counts in KOVA counting chambers). *P value < 0.05.

No strong significant correlation was found between XN and standard method results. The paired Wilcoxon test indicates that the results obtained by the two methods are significantly different. UF and standard method results are strongly correlated and are not significantly different according to the paired Wilcoxon test.

**FIG 2:**
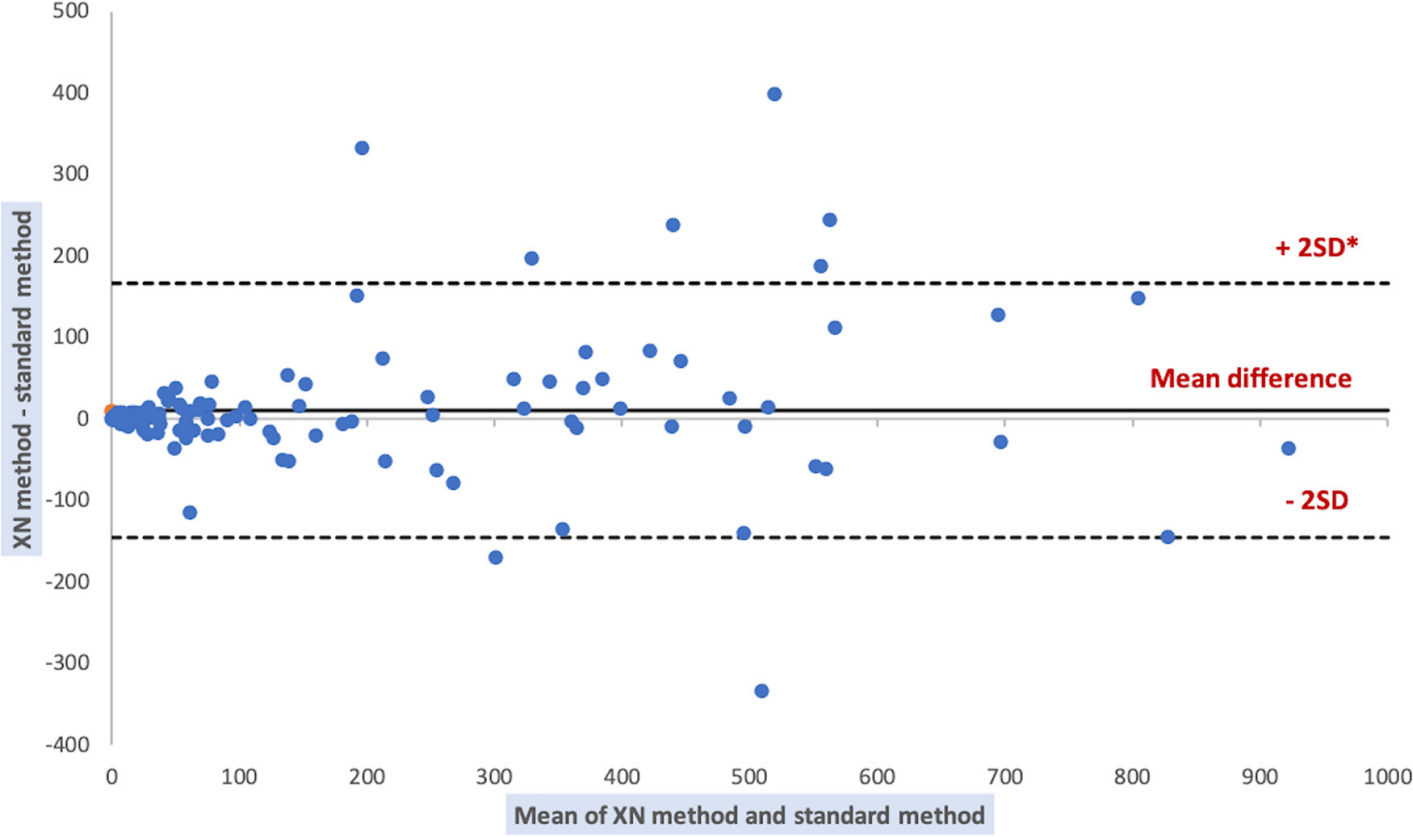
Bland Altman plot of differences between white blood cell counts by XN and standard method. *SD = standard deviation.

**FIG 3:**
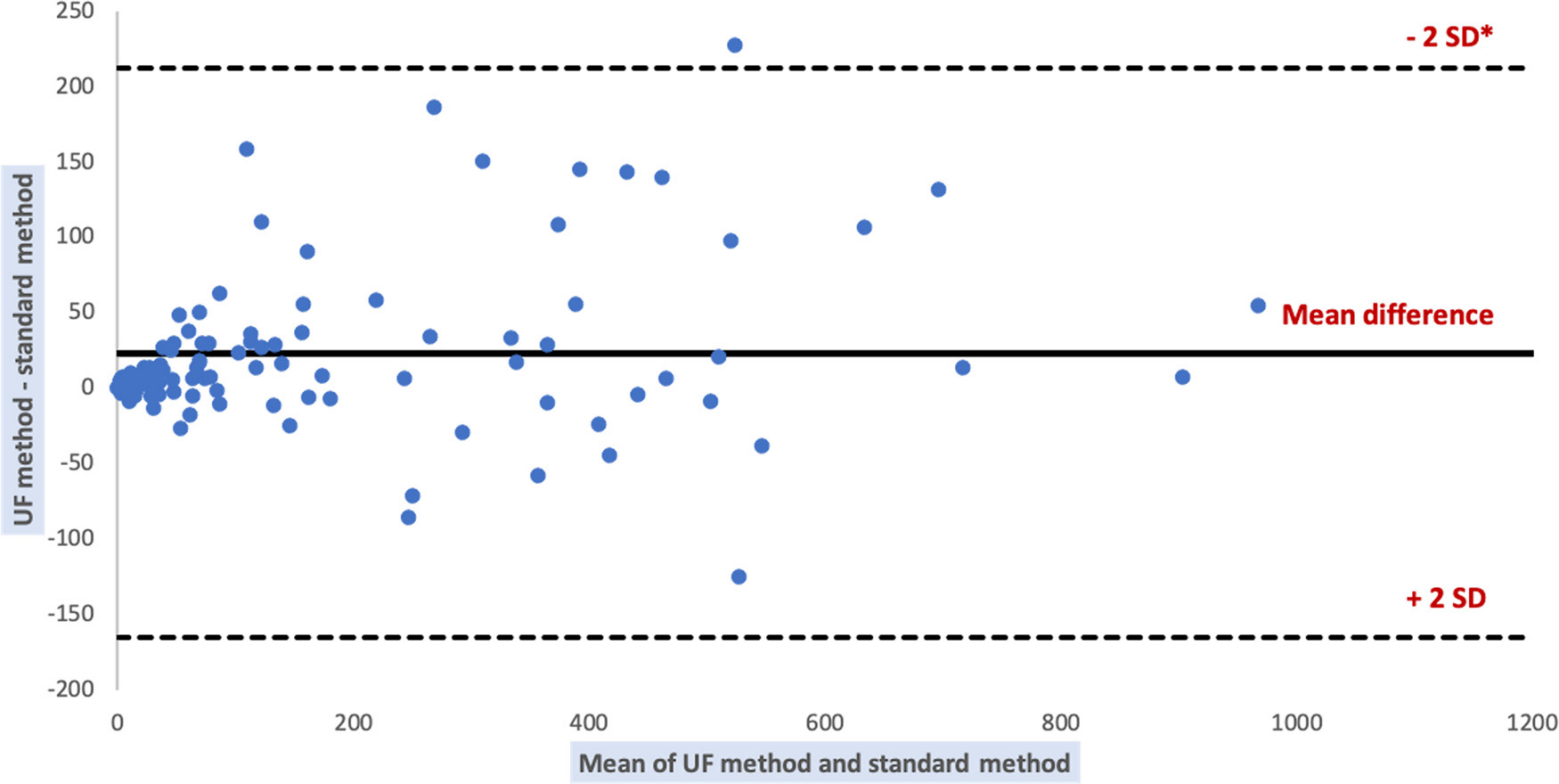
Bland Altman plot of differences between white blood cell counts by UF and standard method. *SD = standard deviation.

Comparison of results of the automated methods and the KOVA standard method for WBC counts are shown in Table 4, Fig. 2, and Fig. 3. The comparison was performed on 135 samples. Forty-seven samples were excluded from the comparative analysis because standard method results were outside the limits of quantification (>1,000 WBC/μL). Two of them had results <1,000 WBC/μL with XN method (840/μL and 915/μL, respectively): one was peritoneal fluid from an organ donor and the other was peritoneal dialysis fluid. Also, two of them had results <1,000 WBC/μL with the UF method (995.7/μL and 454.9/μL, respectively): one was the same peritoneal fluid and the other was a peritoneal dialysis fluid taken from a patient who was on broad-spectrum antibiotherapy.

**TABLE 4:** Comparison of white blood cell counts obtained by XN and UF methods to the standard method^*a*^

Variable	Sysmex XN	Sysmex UF
Mean difference	10.42	23.37
Lower limit of agreement	−145.50	−165.25
Upper limit of agreement	166.34	211.99
Spearman’s correlation coefficient	0.955 (*P* < 0.00001*)	0.9711 (*P* < 0.00001*)
Regression equation	Y = 0.8829x + 8.9227	Y = 0.9734x + 8.444
Paired Wilcoxon test (bilateral)	*P* = 0.112932	*P* = 0.0707247

a All statistical tests were carried out in comparison with standard method (Red blood cell counts in KOVA counting chambers). *P value < 0.05.

Strong and significant correlation was found between XN, UF, and standard method results. The paired Wilcoxon test indicates that the results obtained by the different methods are not significantly different.

**FIG 4:**
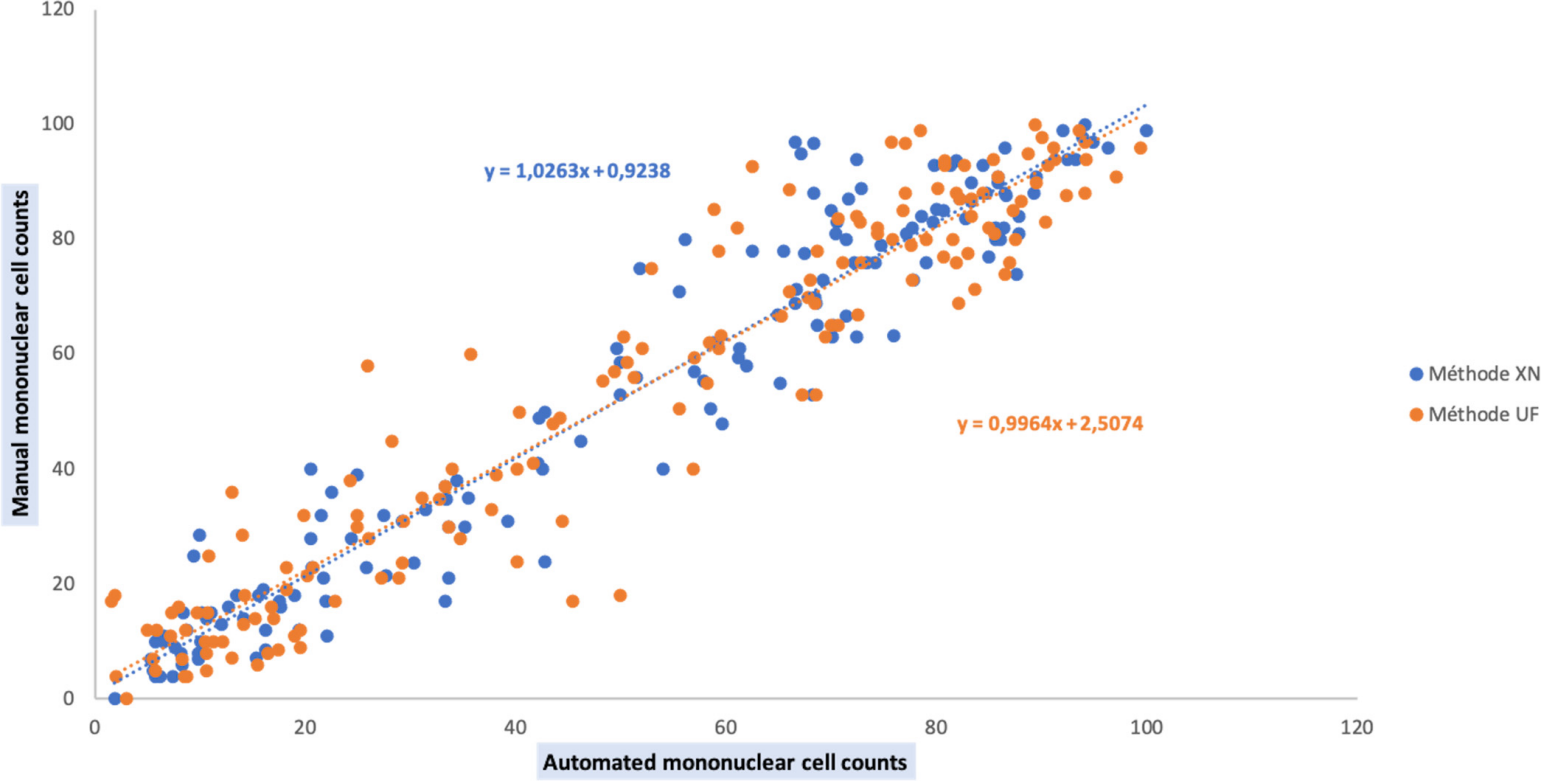
Regression lines between automated (UF et XN) and manual mononuclear cell counts.

**(ii) Differential leukocyte count.** Comparison of the automated methods to the standard method for differential leukocyte counts was performed on 161 biological fluid samples. Twenty-one samples were excluded from the comparative analysis due to an insufficient number of cells on the smear. Results show that the percentage of mononuclear cells obtained by the XN method (*r*^2^ = 0.9027, *P*-value < 0.0001) and by the UF method (*r*^2^ = 0.91, *P*-value < 0.0001) are strongly and significantly correlated with standard method results (Fig. 4).

**FIG 5:**
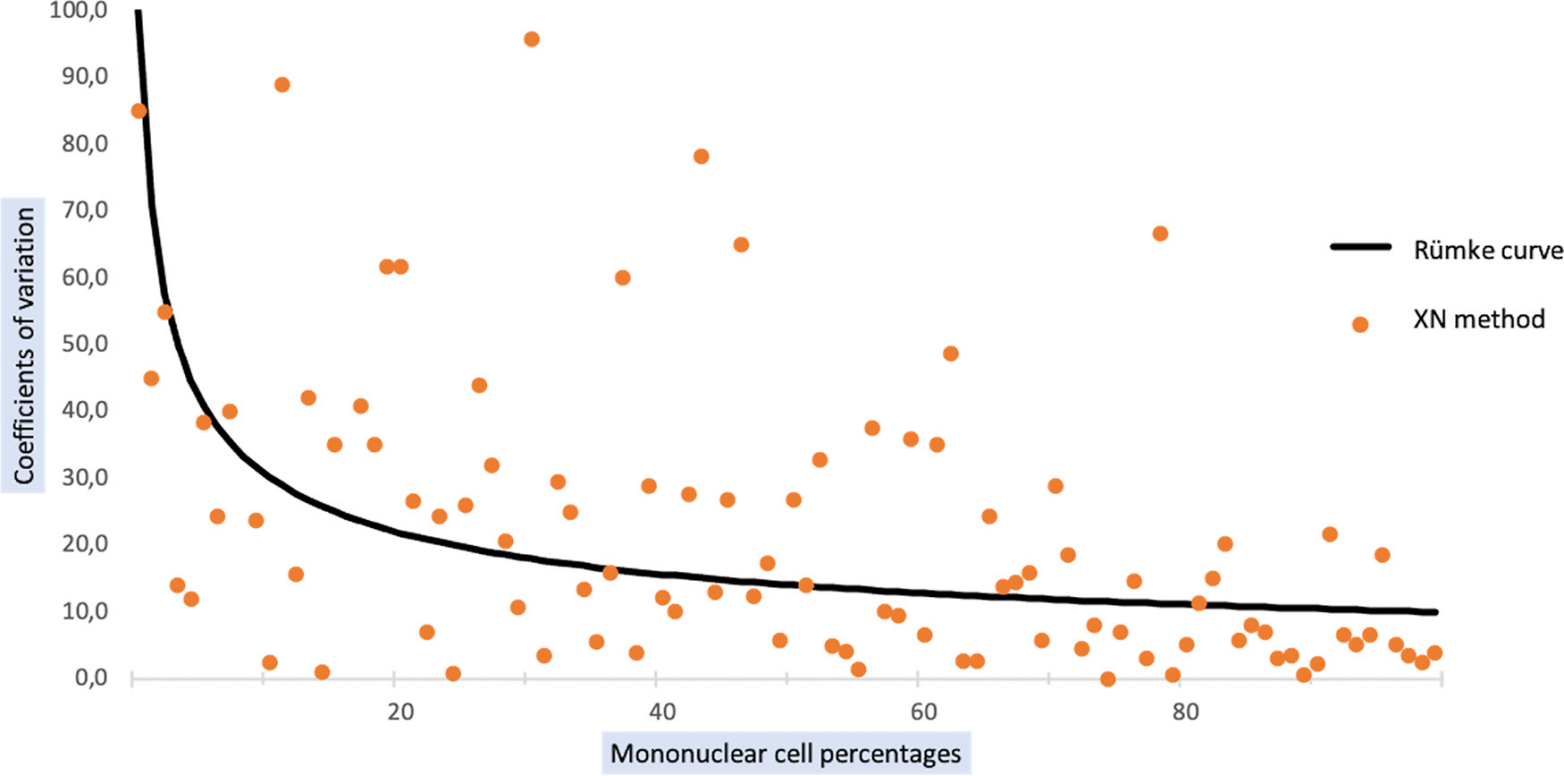
Comparison of mononuclear cell counts obtained by XN and standard methods. The Rümke curve was drawn using the Rümke table; it represents the limit value of acceptable coefficients of variation.

To take into account, the relative variations in leukocyte populations, we used the Rümke table to compare automated and manual mononuclear cell counts. Overall, 60.32% of the values obtained with the XN method lead to acceptable coefficients of variation in comparison with standard method results (Fig. 5).

**FIG 6:**
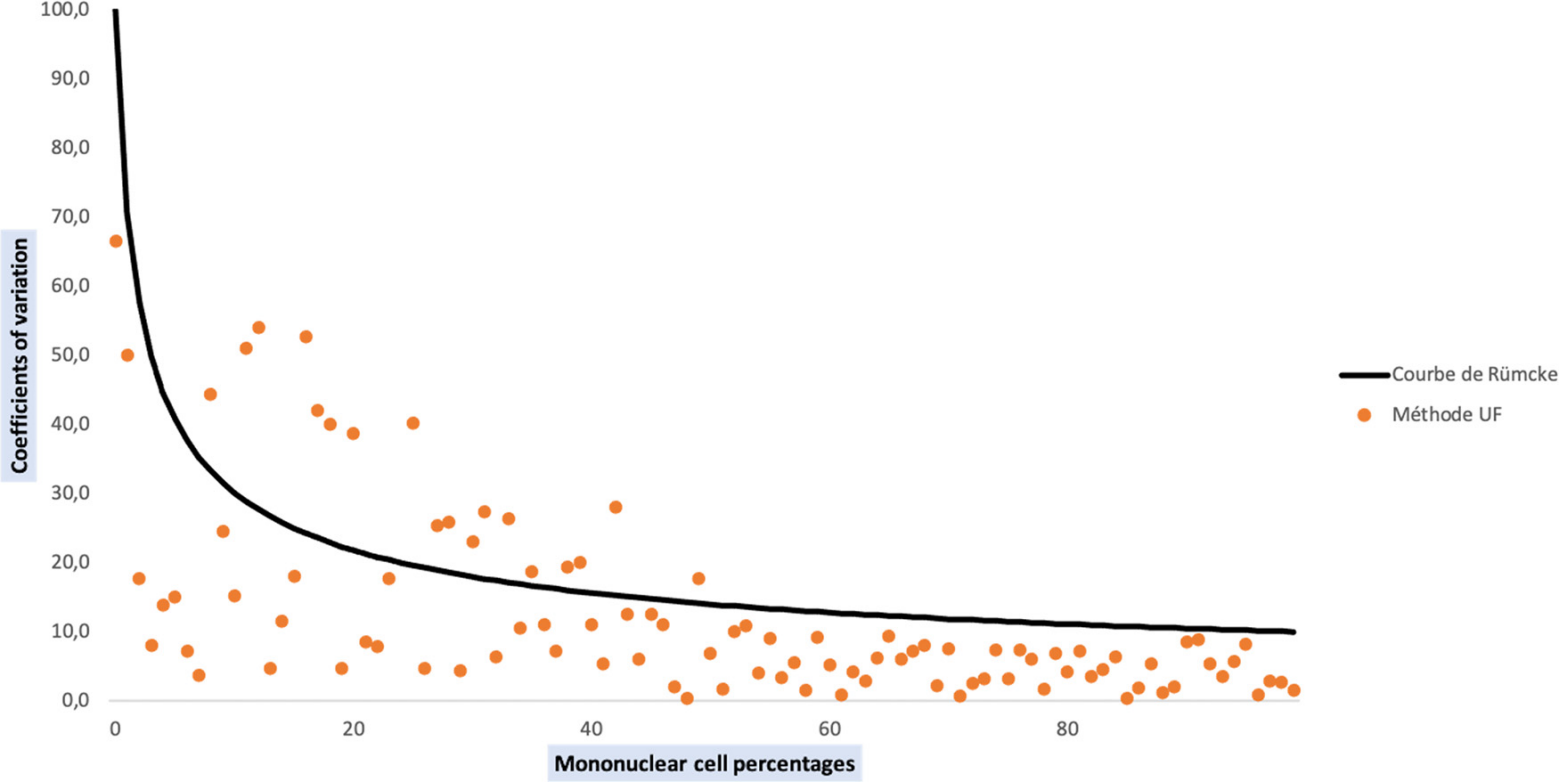
Comparison of mononuclear cell counts obtained by UF and standard methods. The Rümke curve was drawn using the Rümke table; it represents the limit value of acceptable coefficients of variation.

In addition, 84.25% of the values obtained with UF method lead to acceptable coefficients of variation in comparison with standard method results (Fig. 6). Coefficients of variation outside acceptable limits are only observed for low cell counts (percentage of mononuclear cells under 50%). There were 18 samples for which we obtained a coefficient of variation outside of acceptable limits: 7 AF, 5 PF, 2 PRF, 2 CSF, 1 SF and 1 PDF. Among them, 4 AF and 3 PF had abnormal cells on their smear. Both PRF samples had WBC counts above 100,000/μL, and both CSF had WBC counts under 100/μL.

### Comparison of the performance of microbiological analysis methods.

A total of 534 biological fluid samples were analyzed by FCM (bacterial counts) using the SYSMEX UF4000 (Sysmex, Kobe, Japan). They were subsequently processed by routine microbiological procedures. Seven samples were excluded due to a lack of clinical or biological information and 1 sample which was only positive for *Candida* spp. was also excluded. Finally, 526 samples originating from 399 patients were included in this part of the study: 42 AF, 31 PF, 31 PRF, 16 PDF, 281 SF, and 125 CSF. The mean age was 62 years (IQR: 24.5-67) and the sex ratio was 1.21. The characteristics of included patients are shown in Table 5.

**TABLE 5:** Characteristics of included patients for the comparison of the performance of microbiological analysis methods

Variable	All fluids	AF^*a*^	PF^*b*^	PRF^*c*^	PDF^*d*^	SF^*e*^	CSF^*f*^
Total	526	42	31	31	16	281	125
Age (mean ± SD)	62.0 ± 19	64.9 ± 7.8	64.0 ± 17.2	49.4 ± 24.0	51.7 ± 29.3	64.5 ± 19.2	58.0 ± 17.9
Sex ratio	1,21	2,23	1,14	1,38	1,29	1,30	0,84
Direct positive Gram staining, *n* (%)	66/512 (12.89%)	2/42 (4.76%)	0/31 (0.00%)	10/31 (32.26%)	1/16 (6.25%)	47/272 (17.28%)	6/120 (5.00%)
Positive culture *n* (%)	98/505 (19.41%)	3/29 (10.34%)	1/28 (3.57%)	16/31 (51.61%)	5/14 (35.7%)	60/278 (21.58%)	13/125 (10.40%)
Polymicrobial culture *n* (%)	11/98 (11.22%)	1/3 (33.33%)	0/1 (0.00%)	8/16 (50.00%)	0/5 (0.00%)	5/60 (8.33%)	0/13 (0.00%)

a AF: ascitic fluid.

b PF: pleural fluid.

c PRF: peritoneal fluid.

d PDF: peritoneal dialysis fluid.

e SF: synovial fluid.

f CSF: cerebrospinal fluid shunt.

Table 6 shows the median and IQR values of bacteria and WBC counts estimated by FCM of samples included according to culture or DGS positivity. Bacteria and WBC counts values estimated by FCM are significantly higher for samples with a positive culture than those with a negative culture. In the same way, bacteria and WBC counts values estimated by FCM are significantly higher for samples with a positive DGS than those with a negative DGS.

**TABLE 6:** Median and interquartile ranges (IQRs) of flow cytometry parameters according to culture positivity and direct Gram staining

Variable	Bacteria/μL			WBC/μL		
	Median	IQR	*P*	Median	IQR	*P*
Positive culture	382	64.65–2082.5	*P* < 0.0001^*a*^	3062.8	242.8–8757.75	*P* < 0.0001^*a*^
Negative culture	43	15–124.85		99.9	29.3–626.9	
Direct positive Gram staining	635	190–3734.30	*P* < 0.0001^*a*^	3595	916.75 – 10096	*P* < 0.0001^*a*^
Direct negative Gram staining	43	15.8–126.25		100.4	29.5 – 651	

a Mann-Whitney.

DGS was performed in 512 samples, but culture was performed in only 505 samples (98.44%). In this study, comparing DGS results to culture results, it was found that DGS has a sensitivity of 50.00% and a specificity of 99.24% according to the culture results for the total of samples of our study (Table 7).

**TABLE 7:** Sensitivity and specificity of Direct Gram staining according to culture results

Variable	All fluids	PRF^*a*^	SF^*b*^	CSF^*c*^
Sensitivity (%)	50.00	62.5	51.67	41.67
Specificity (%)	99.24	100	99.52	99.07

a PRF: peritoneal fluid.

b SF: synovial fluid.

c CSF: cerebrospinal fluid shunt.

The ROC curves of bacteria/μL and DGS positivity yielded an area under the curve (AUC) of 0.97, 0.88 and 0.89 for PRF, SF and CSF, respectively. The ROC curves for PDF, AF and PF were not calculated due to the low number of PDF included and because of the low number of positive samples among AF and PF (Table 8).

**TABLE 8:** Areas under the curve and optimal cutoff points for bacterial count by flow cytometry versus direct Gram staining positivity and culture positivity

Variable	Performance characteristics	All fluids	PRF^*a*^	SF^*b*^	CSF^*c*^
Direct Gram stain	Area under the curve	0.88	0.97	0.88	0.89
	Optimal cutoff point (bacteria/μL)	185.0	465.0	1200.0	17.2
	Sensibility/specificity (%)	77.8/82.5	100/85.7	95.7/67.1	100/54.4
	Positive predictive value/negative predictive value (%)	42.1/95.8	76.9/100	37.8/98.7	10.3/100
Microbial culture	Area under the curve	0.83	0.83	0.80	0.60
	Optimal cutoff point (bacteria/μL)	465.0	541.7	6340.0	57.0
	Sensibility/specificity (%)	58.2/96.3	62.5/93.33	45.0/93.6	46.2/83.0
	Positive predictive value/negative predictive value (%)	79.2/96.5	90.9/70.0	65.9/86.1	24.0/93.0

a PRF: peritoneal fluid.

b SF: synovial fluid.

c CSF: cerebrospinal fluid shunt.

**FIG 7:**
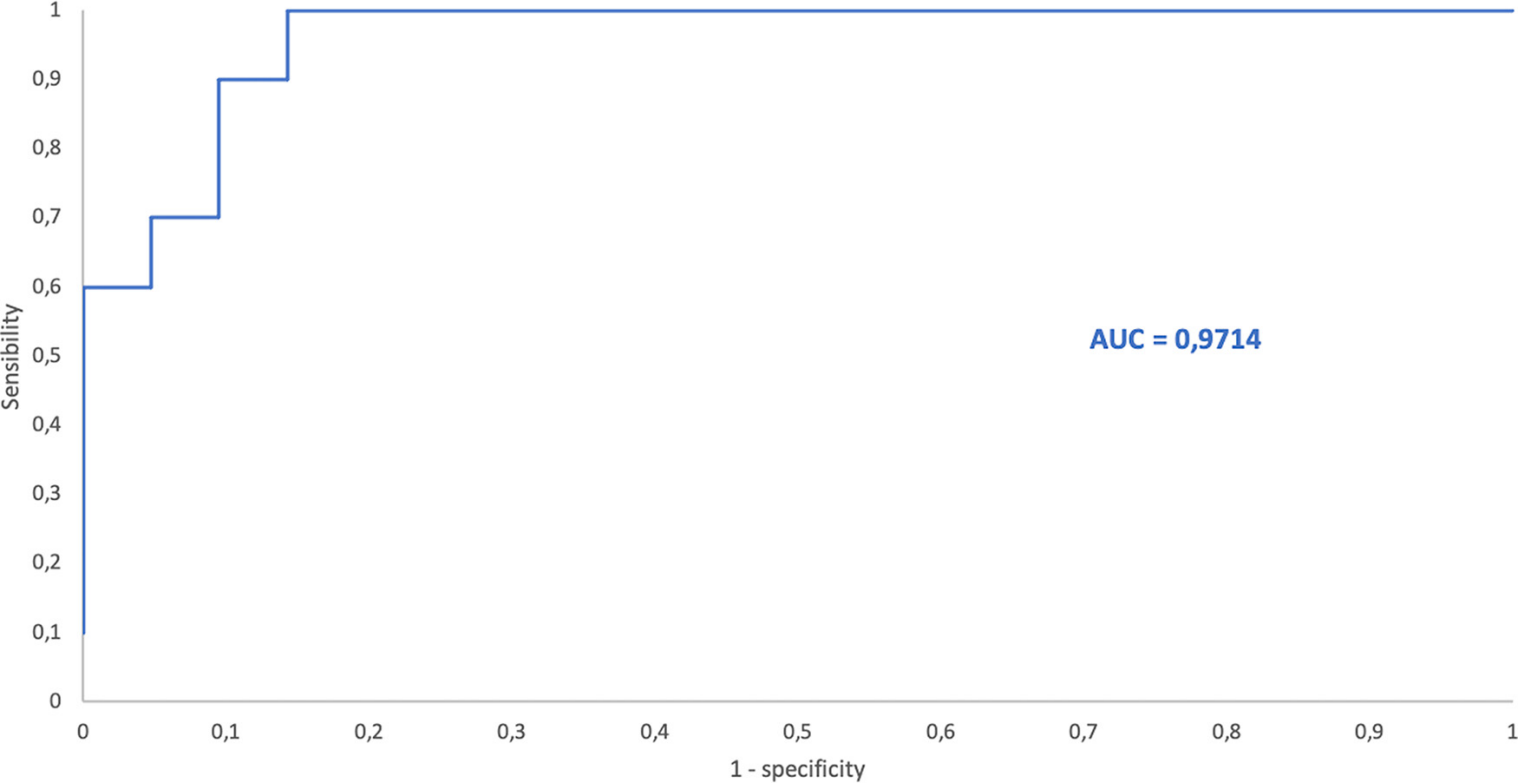
ROC curve for bacterial counts by FCM versus DGS positivity for peritoneal fluid samples. AUC = area under the curve.

Applying the calculated cutoff point (465.0 bacteria/μL) to the PRF samples, all PRF samples with DGS positive results were obtained (Fig. 7). Also, 3 PRF samples with bacterial counts over the cutoff value by FCM (1035.4, 712.7 and 496, respectively) and DGS negative results were obtained. The first had a polymicrobial culture (E. coli, P. mirabilis, and anaerobic bacteria), while the second had a WBC count of 54402/μL and was taken from a patient with acute appendicitis who was under antimicrobial treatment (3rd generation Cephalosporin + Metronidazole) when the sample was obtained. The third had a WBC count of 7146.7/μL and was taken from a patient with peritonitis who was under antimicrobial treatment (3rd generation Cephalosporin + Metronidazole) when the sample was obtained. For these three patients, a final diagnosis of infection was established based on clinical symptoms and the infectious etiology was confirmed by other samples (blood cultures or other biological fluids).

**FIG 8:**
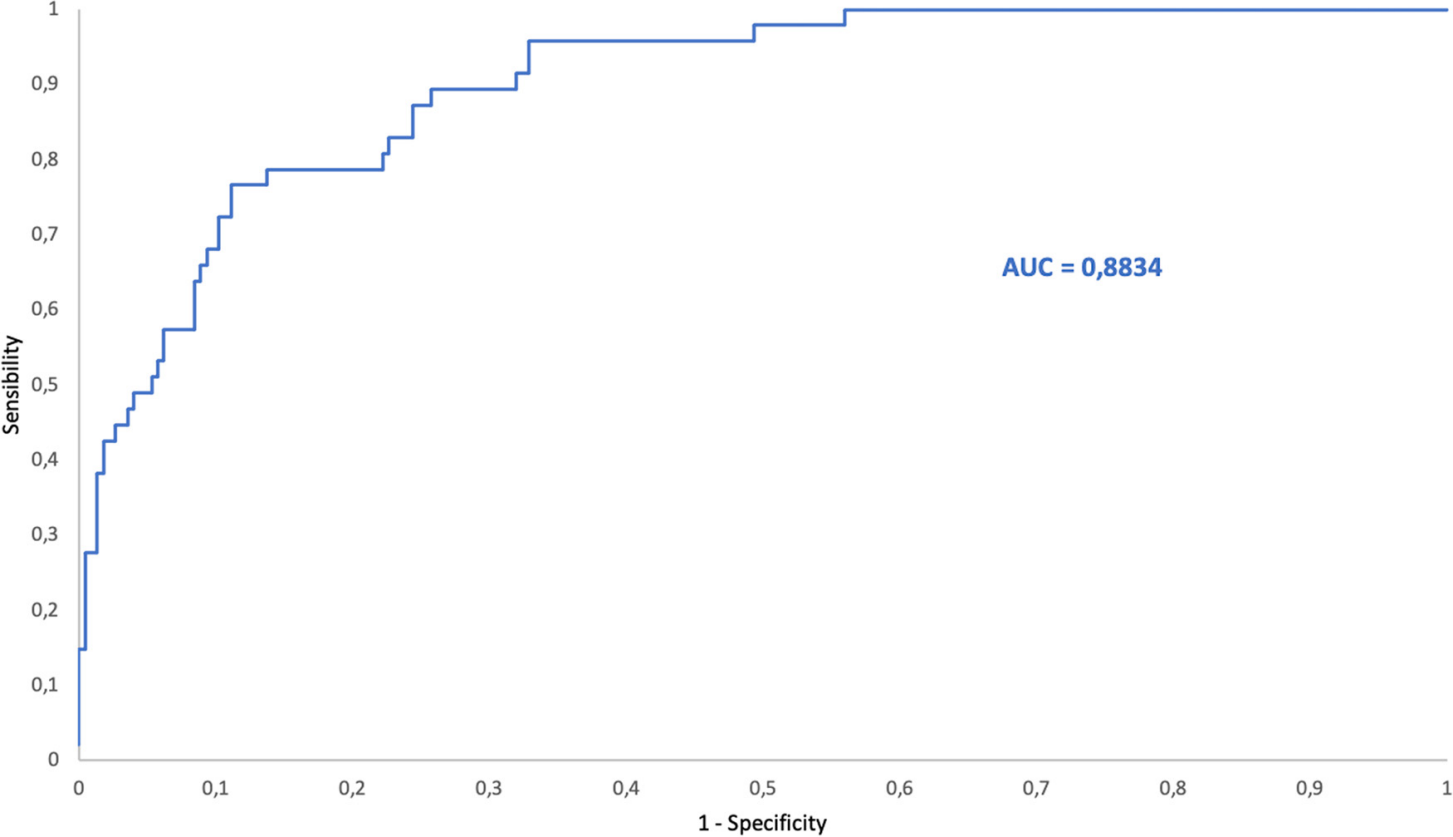
ROC curve for bacterial counts by FCM versus DGS positivity for synovial fluid samples. AUC = area under the curve.

Applying the calculated cutoff point (1,200.0 bacteria/μL) to the SF samples, 45 of 47 SF samples with DGS-positive results were obtained (Fig. 8). In the two patients with samples showing bacterial counts under the cutoff point and DGS positive results, a final diagnosis of infection was not established. Both had Gram positive cocci in DGS, but one had culture negative result and the other one had culture positive result (S. capitis). We suspected a false positive DGS result for the first and contamination of the microbial culture for the second. Seventy-four samples that showed high bacterial counts by FCM had DGS-negative results. Among these 74 samples, 9 were taken from patients with a final diagnosis of infection (established based on clinical symptoms). Moreover, two of them had culture positive results (S. marcescens and S. lugduniensis, respectively).

**FIG 9:**
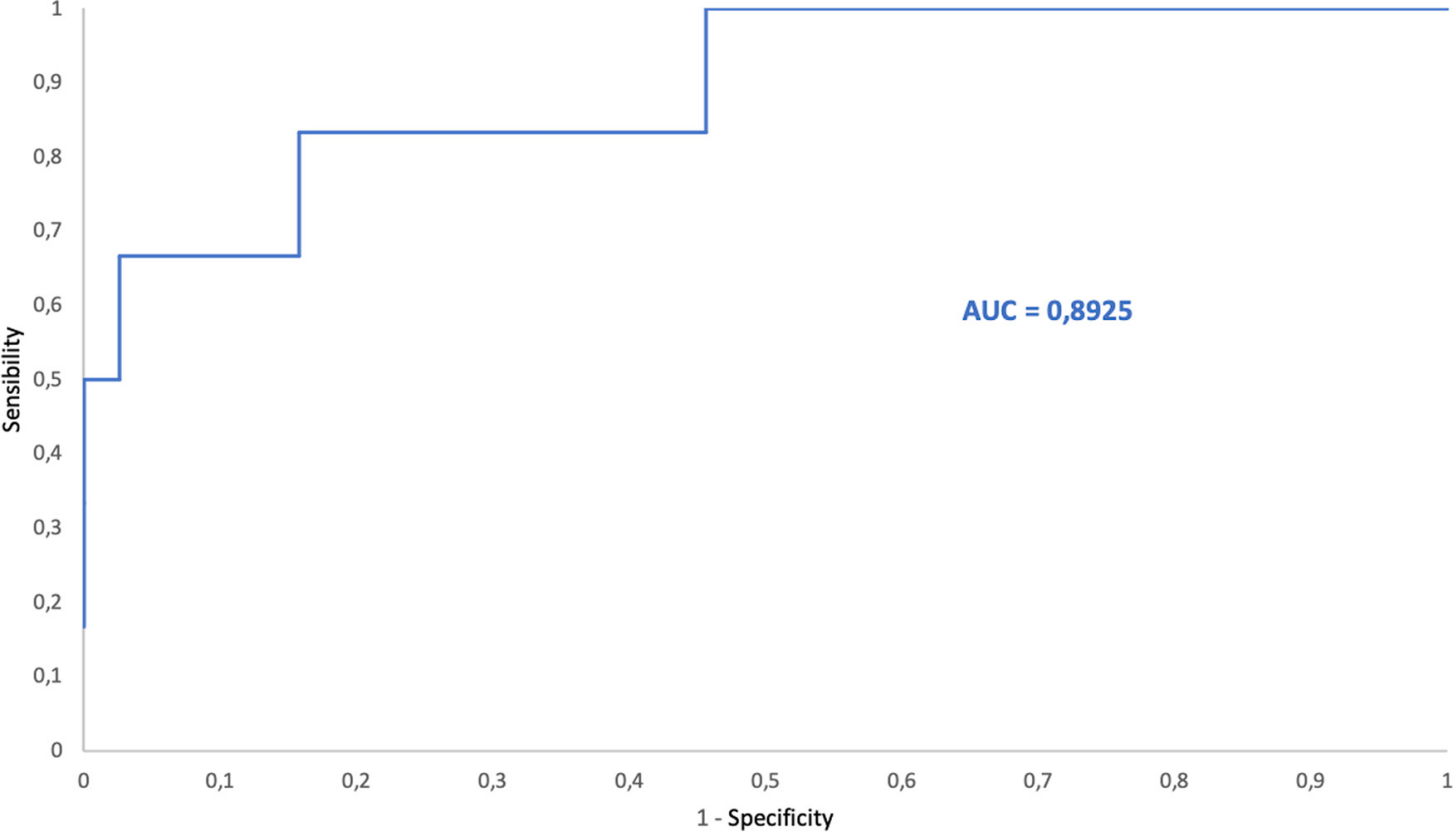
ROC curve for bacterial counts by FCM versus DGS positivity for cerebrospinal fluid samples. AUC = area under the curve.

Applying the calculated cutoff point (17.2 bacteria/μL) to the CSF samples, all CSF samples with DGS-positive results were obtained (Fig. 9). Fifty-two samples with high bacterial counts by FCM had DGS-negative results; six of them were taken from patients with a final diagnosis of infection (established based on clinical symptoms) and the infectious etiology was confirmed on other samples (other CSF samples). Among these 6 samples, 3 had culture-positive results (S. aureus, S. hominis, and S. epidermidis, respectively).

## DISCUSSION

Cytobacteriological analysis of biological fluids is crucial in the management of patients suffering from effusions or infectious diseases. It requires cytological expertise for performing RBC counts, WBC counts, and differential leukocyte counts. It also requires microbiological expertise for performing DGS and for the interpretation of microbial cultures. We evaluated the flow cytometer UF4000 (Sysmex, Kobe, Japan) as a cytological and bacteriological analysis method in different biological fluid samples.

We compared the performance of cytological analyses of the UF4000 (Sysmex, Kobe, Japan) with the performance of the XN10 flow cytometer (Sysmex, Kobe, Japan) as well as that of standard reference methods (manual cell counts in KOVA counting chambers and manual differential leukocyte counts). With regard to cell counts, UF4000 (Sysmex, Kobe, Japan) showed a performance which was at least equivalent to those of the reference methods and superior to those of XN10 (Sysmex, Kobe, Japan). The Bland Altman plot of differences between RBC counts showed an average bias of -15.16/μL for the UF method. It is a weak bias, especially because there is no diagnostic threshold value for RBC counts. The appearance of the sample (haemorrhagic or not) is sufficient to allow a diagnostic orientation. An acceptable average bias was obtained for WBC counts (23.37/μL). Regarding the differential leukocyte counts, UF4000 (Sysmex, Kobe, Japan) showed excellent performance for body fluids which did not contain abnormal cells. Our results agree with those of other studies, having demonstrated that the FCM allows to correctly classify and enumerate WBCs in the CSF, PRF and PDF (5–10). Because of its characteristics, the XN10 (Sysmex, Kobe, Japan) is suitable for the analysis of samples containing a large quantity of RBC and WBC (>10^3^/μL). Therefore, the UF4000 (Sysmex, Kobe, Japan) seems more suitable for the cytological analysis of biological fluids which most often have low cell counts. However, it does not allow the differentiation of different types of leukocytes (lymphocytes, monocytes, polymorphonuclear neutrophils, eosinophils, polymorphonuclear basophils) and the detection of abnormal cells, which always makes it essential to carry out a manual smear for PF and AF. The main limitation of our study is the quantity of fluid sample necessary to perform the FCM analysis. Samples with a volume of less than 1 mL were systematically excluded (data not recorded), which limited our capacity for inclusion. Also, the standard method and flow cytometers had different limits of quantification and linearity ranges, which limited our ability to compare performance for body fluid samples with RBC or WBC counts with extreme values. Indeed, the automated methods had high limits of quantification which were markedly higher than those of the reference method.

Microbiological analysis results in biological fluids are tools for the management of patients suffering from infectious diseases. These results are crucial for the administration of targeted antimicrobial treatment and management of the infection. Requiring an incubation period, the results of microbial cultures can often only be obtained at least 24 h after taking the sample; hence, the need for rapid detection methods for bacteria in biological fluid samples. We evaluated the performance of the flux cytometer UF4000 (Sysmex, Kobe, Japan) as a method for the rapid detection of bacteria in a variety of biological fluid samples and compared it with the culture and DGS results. There was a consensus between the bacterial count obtained by FCM and, DGS and culture results. Biological fluid samples with DGS-positive results had significantly higher bacterial and WBC counts than samples with DGS-negative results. Also, samples with culture-positive results had significantly higher bacterial and WBC counts than samples with culture-negative results. Our results are in agreement with previous studies which have demonstrated that bacterial count by FCM showed a good correlation with culture results (6, 8, 10, 11). We established optimal cutoff points for bacterial count by FCM in order to obtain DGS and culture-positive results. Several studies have suggested replacing the microbial culture with FCM for urine samples (12–14). According to our results, biological fluid samples with low bacterial counts had a low probability of being culture-positive (NPV = 96.53%). However, due to the number of false-negative samples, the kind of patients involved, the precious nature of the samples and the potential severity of infections, the cessation of culture could not be considered in this type of sample. However, in order to determine the potential interest of FCM to replace DGS, we chose to determine specific and optimal cutoff points for each type of biological fluid sample so that they are as appropriate as possible. We have established three cutoff points for PRF (465.0 bacteria/μL), SF (1,200.0 bacteria/μL) and CSF (17.2 bacteria/μL) with maximum sensitivity and negative predictive values. Unlike the DGS, the UF4000 flow cytometer (Sysmex, Kobe, Japan) does not provide an indication of the type of bacteria detected (Gram+, Gram-, cocci, bacilli). FCM is an automated and reliable method that could be used upstream of routine microbiological procedures. DGS may not be performed on biological fluid samples with bacterial counts below the defined cutoff points. In our study, this would have represented a total of 233 (47.3%) samples (18 PRF [58.1%], 153 SF [55.0%], and 62 CSF [51.7%]) for which the FCM could have replaced the DGS. This suggests that automating the bacteriological analysis of biological fluid samples could save significant technical time without losing efficiency. This work must be continued by other studies which will include greater numbers of fluid samples (especially PF, AF, and PDF) to be able to determine optimal and specific cutoff points. In addition, the performance of mycological analyses of the UF4000 (Sysmex, Kobe, Japan) should be evaluated. Among the samples analyzed, only one had DGS and culture-positive results with C. albicans; it was excluded from the study. This was an SF sample with WBC counts of 101,920/μL and “bacterial” counts above 1,000,000/μL. Therefore, it would be interesting to continue this work in order to evaluate the performance of the UF4000 (Sysmex, Kobe, Japan) as a method for the detection of yeasts in biological fluid samples.

To the best of our knowledge, this is the first study to evaluate the performance of the UF4000 (Sysmex, Kobe, Japan) for the detection of bacteria in different types of biological fluid samples.

These results are promising for the organization of microbiology laboratories and sampling circuits. The possibility of carrying out cytological and microbiological analyses of biological fluid samples on the same automated machine would simplify the sampling circuit (addressing the sample in a single laboratory, 24/7). It would also minimize the quantity of sample required (1 mL for all of these analyses). However, the technical team will retain added value as cytological expertise for the search of abnormal cells or microbiological expertise for characterization of the type of bacteria detected. A “flag” when detecting abnormal cells on the UF4000 (Sysmex, Kobe, Japan) is being investigated by the supplier.

In conclusion, the bacterial count by FCM according to the UF4000 (Sysmex, Kobe, Japan) correlates with DGS and culture results. It could be used upstream of routine microbiological procedures to improve and accelerate the diagnosis of infection in biological fluid samples.

## MATERIALS AND METHODS

### Sample inclusion.

In total, 538 biological fluid samples received at the Microbiology or Haematology Departments of Caen University Hospital between June 2020 and July 2021 were included in the study. The samples were collected in sterile tubes with no chemical preservatives and processed immediately after arrival.

**Comparison of the performance of cytological analysis methods.** Six different kinds of biological fluids were processed by the routine cytological procedures and subsequently by the SYSMEX XN (Sysmex, Kobe, Japan) and the SYSMEX UF4000 (Sysmex, Kobe, Japan): AF, PF, PRF, PDF, SF, and CSF. The SF was previously diluted in saline solution before analysis (1:10 dilution). Specimens with insufficient volume (less than 1 mL) were excluded (data not recorded).

Some clinical and biological information was sought in the patient files: medical reason for the puncture of biological fluids and reported history of hematological malignancy.

**Comparison of the performance of microbiological analysis methods.** Six different kinds of biological fluids were processed by the routine microbiological procedures and subsequently by the SYSMEX UF4000 (Sysmex, Kobe, Japan): AF, PF, PRF, PDF, SF, and CSF. The SF was previously diluted in saline solution before analysis (1:10 dilution). Specimens with insufficient volume (less than 1 mL) were excluded (data not recorded).

Some clinical and biological information was sought in the patient files: clinical symptoms, anti-infectious treatment and diagnosis of infection.

### Standard methods.

**(i) Routine cytological procedures.** Cell counting (red blood cells and leukocytes) of body fluids was done manually, using 10 cell count slides with grids (KOVA Glasstic Slide 10 [CML, Nemours, France]). The cells were quantified under ×10 and ×40 magnifications. The results were reported as the number of cells per microliter.

The differential leukocyte count of the body fluids was done manually after making a spot with cytospin and then staining with MGG. The differential count was performed at ×100 magnification on a total count of 100 cells with the differentiation of polynuclear cells and mononuclear cells.

**(ii) Routine microbiological procedures.** A DGS was performed in centrifugation pellets (10 min at 3,000 rpm) for all PRF, PF, SF, and, in bloody and/or mucous PDF. For non-mucous LDP, the DGS was performed after making a cytospin spot (two drops, 5 min). A DGS on the primary sample was performed for all AF and CSF with sufficient volume and with leukocyte count greater than or equal to 10/mm^3^.

Biological fluids were inoculated manually. PDF and AF were added into aerobic and anaerobic blood cultures bottles. These bottles were incubated into the BacT/Alert VIRTUO (bioMérieux, Marcy-L’étoile, France) for 7 days. CSF were inoculated on chocolate agar (bioMérieux, Marcy-L’étoile, France) and on Brain Heart Infusion medium (bioMérieux, Marcy-L’étoile, France). PF were inoculated on blood agar (bioMérieux, Marcy-L’étoile, France), chocolate agar (bioMérieux, Marcy-L’étoile, France) and on Schaedler medium (bioMérieux, Marcy-L’étoile, France). PRF were inoculated on blood agar (bioMérieux, Marcy-L’étoile, France), chocolate agar (bioMérieux, Marcy-L’étoile, France), Bromocresol purple agar (bioMérieux, Marcy-L’étoile, France), Schaedler medium (bioMérieux, Marcy-L’étoile, France), and on selective chocolate agar TM+PolyViteX VCAT3 (bioMérieux, Marcy-L’étoile, France) for female patient. SF were inoculated on chocolate agar (bioMérieux, Marcy-L’étoile, France), blood agar (bioMérieux, Marcy-L’étoile, France) and incubated on blood cultures bottles into the BacT/Alert VIRTUO (bioMérieux, Marcy-L’étoile, France) for 14 days. Bacterial identification was achieved using MALDI-TOF MS (Matrix associated laser desorption/ionization time-of-flight mass spectrometry) (Bruker, Bremen, Germany).

### Automated methods compared.

**(i) Sysmex UF4000 (Sysmex, Kobe, Japan).** SYSMEX UF4000 (Sysmex, Kobe, Japan) is an automated urine and body fluid analyzer whose technology is based on flow fluorocytometry with a blue laser. It provides 9 parameters for body fluids: erythrocyte count (RBC), total nucleated cell count (TNC), epithelial cell count, white blood cell count (WBC), count and percentage of mononuclear cells (MN#, MN%), count and percentage of polynucleated cells (PN#, PN%), and the count and classification of bacteria (BACT). Counting and classification is performed in three steps: cell labeling (nucleic acids, membrane components or surface proteins), hydrodynamic focusing and finally characterization by the blue laser using four different signals. The FSC, SFL, SSC and DSS signals, respectively, provide information for particle size, fluorescence, internal complexity and depolarization.

All samples were analyzed by flow cytometry using the Sysmex UF4000 (Sysmex, Kobe, Japan) and following the manufacturer’s recommendations. Samples were analyzed manually using the STAT mode and the required minimum sample volume was 0.6 mL. High and low positive controls were processed twice daily.

**(ii) Sysmex XN (Sysmex, Kobe, Japan).** Sysmex XN (Sysmex, Kobe, Japan) is an automated blood samples analyzer. It is made of different modules. The classic module performs blood counts. The expert modules perform fluorescence counts of blood platelets and reticulocytes. The “Body fluid” module performs blood fluid samples analysis by using two channels. The RET channel performs red blood cell counts by impedance. The WDF channel performs white blood cell counts by flow fluorocytometry. It provides different parameters: total nucleated cell (TC-BF), white blood cell count (WBC-BF), percentage of mononuclear cells, and percentage of polynucleated cells.

Samples were analyzed on the SYSMEX XN (Sysmex, Kobe, Japan) after their analysis on the SYSMEX UF4000 (Sysmex, Kobe, Japan) following manufacturer’s recommendations. Samples were analyzed manually on the “body fluid” module. The required minimum sample volume was 0.3 mL. Controls were processed twice daily.

### Statistical analysis.

Statistical analyses was performed using EXCEL and XL STAT.

**Comparison of the performance of cytological analysis methods.** Red and white blood cell counts were compared using the Spearman’s correlation coefficient and the non-parametric Wilcoxon test for the comparison of two dependent variables.

Differential leukocyte counts were compared using the Spearman’s correlation coefficient and the Rümke table. The differences were considered statistically significant with a *P*-value < 0.05.

**Comparison of the performance of microbiological analysis methods.** The different parameters measured by FCM were defined as dependent variables using the culture and DGS results as independent variables. The distribution of independent variables across groups was compared using the Mann-Whitney U test for the comparison of two independent variables. The differences were considered statistically significant with a *P*-value < 0.05.

The receiver operating characteristic (ROC) curves were used to compare the UF4000 bacterial counts to the culture and DGS results obtained by routine procedures. The cutoff values were chosen based on best balance between sensitivity and specificity, giving priority to sensitivity in order to detect DGS-positive samples and giving priority to specificity in order to detect culture positive samples.

### Ethical statement.

The study was carried out without any additional intervention in patients. All samples were processed following routine cytological and microbiological procedures. Patient data were anonymized before analysis. Demographic, clinical, and biological data were obtained from the laboratory information system (TD NEXLABS) and the hospital information system (REFERENCE).
